# Could an Open-Source Clinical Trial Data-Management System Be What We Have All Been Looking For?

**DOI:** 10.1371/journal.pmed.0050006

**Published:** 2008-03-04

**Authors:** Greg W Fegan, Trudie A Lang

## Abstract

The authors argue that research organizations and funders should combine efforts to produce an open-source solution for trial data management.

## Difficulties in Meeting the Demands of Regulators and Guidelines

In Europe, it is a legal requirement to conduct clinical trials in accordance with the International Conference on Harmonisation's guidelines on good clinical practice (see http://www.ich.org/). A recent editorial reported that this directive has led to a decline in the number of trials being conducted by independent academic groups [1]. One possible reason for this is that reporting and documentation requirements are now so burdensome that the process has become unnecessarily complicated [2]. This is rather ironic, given that well-designed clinical trials should be amenable to very simple data handling and analysis [3]. Indeed the flowchart established by the CONSORT (Consolidated Standards of Reporting Trials) statement [4] for carrying out a properly randomised controlled trial has just four steps, which supports the approach of keeping it simple.

Following discussions with colleagues at various institutions (including Oxford University, the London School of Hygiene and Tropical Medicine, the International Aids Vaccine Initiative, and the Medical Research Councils of Uganda, South Africa, and the United Kingdom), one major difficulty comes up time after time: these, and many other, clinical trial groups do not have the skills or resources to establish and use software systems required to manage trial data in compliance with the International Conference on Harmonisation's guidelines. This situation is further exacerbated for non-commercial research groups based in developing countries, where basic information systems infrastructure and support tends to be even more limited [5].

There is little good independent information about what is available. Additionally, there is almost a complete absence of guidance from regulatory agencies such as the European Medicines Agency and United States Food and Drug Administration about how to evaluate the many competing systems available, and indeed what the actual requirements are for trials where the data will be needed for a regulatory submission. This is particularly important with respect to trials evaluating products for neglected diseases, which are often carried out by academic researchers and where the data would be needed to support a product license. The size of this issue can be somewhat ascertained from the results of a search that we did at the World Health Organization trials registration site (http://www.who.int/trialsearch/, accessed September 27, 2007): use of the term “Africa” returned 206 trials, and the term “Asia” returned 520.

Ideally, such a system would work as well for a single-centred investigator-led small trial as it would for large regulatory standard multi-centred randomised controlled trials. Furthermore, this system would need to be affordable to the public sector and modifiable and amenable for use with existing software already employed, particularly statistical and reporting software. This is quite a tall order. Put in this context, and considering the dialogue between research groups on this matter, it would seem prudent for international health research organisations to combine their efforts and spending power and assist with the development of systems that are open to all and truly fit for purpose. The daunting challenges of capturing, cleaning, extracting, and storing trial data would then be eased, with the added desirable benefit of improving quality and reliability of data. Perhaps we would then see more academics wanting to conduct clinical research.

## The Cost, Complexity, and Availability of Current Systems

It has been noted [6] that clinical trials–related software can be prohibitively expensive, especially for individual researchers, or groups based in developing countries. The two most commonly used packages [7], Oracle Corp's Oracle Clinical (http://www.oracle.com/industries/life_sciences/oracle-clinical.html) and Phase Forward's Clintrial (http://www.phaseforward.com/products/clinical/cdm/cis/), are both designed for use with commercial database systems. Investing in such systems would cost in the range of hundreds of thousands of dollars, depending on the size of the trial and number of licenses needed. Such costs would take up a disproportionate amount of a typical non-commercial trial budget, which is generally in the same order of magnitude as the cost of these systems, and must cover everything required by the trial. This leads many developing country institutions with the unenviable choice of either not being able to comply with international standards or having to send case report forms off-site for processing. This lamentable state of affairs has been acknowledged by some funding groups, such as the Gates Foundation–supported Malaria Clinical Trials Alliance [8], the European and Developing Countries Clinical Trials Partnership, and the African Malaria Network Trust; however, addressing the need for an affordable, easy-to-use clinical trial data-management system is currently beyond the scope and remit of the capacity-building activities being rolled out by such groups.

## An Early Attempt to Address the Gap

Cynthia Brandt and Prakash Nadkarni of the Yale Center for Medical Informatics, with their TrialDB system (http://ycmi.med.yale.edu/trialdb/), have championed a non-commercial approach since the 1990s [9]. However, although their software is freely available, its use is targeted to either Oracle's Database Management System or Microsoft's SQL Server for case report form generation and data capture and management. Whilst this “free” system is a good starting point for those who may already have the appropriate licensing and expertise for the required commercial components, its use in resource-poor countries and by individual researchers is likely to be limited. However, it is not inconceivable that this application could be re-engineered to take advantage of the free (for non-commercial use) and open-source database systems such as MySQL (http://www.mysql.org/) and PostgreSQL (http://www.postgresql.org/). Such an initiative could be funded by international health agencies for minimal outlay. Just as funders of biomedical research are starting to require scientific output to be published in open-access journals, could they not require that the software used for the management of clinical trials also be open? Even for commercial research organisations, such an approach can only be to their longer-term benefit, given the likely savings they would incur due to reduced software costs.

## A Way Forward?

We propose a commitment by the major international donor and implementing groups to encourage efforts to develop a free and open-source data-management system for clinical trials that adheres to evolving standards such as those set by CDISC (the Clinical Data Interchange Standards Consortium; http://www.cdisc.org). We believe that an open-source approach has the best chance of ensuring that all kinds of groups can be involved with the development of systems that have bearing on global public health.

The US National Institute of Health's National Cancer Institute has a wide-ranging, quickly evolving, and very open-source friendly [10] initiative called CaBIG (Cancer Biomedical Informatics Grid), which includes clinical trials management systems, amongst others (see [11]). One of the many projects involved in CaBIG, OpenClinica (http://www.openclinica.org/), has used CaBIG as a springboard to launch and maintain a free and open-source clinical trials data-management system. This software is entirely built using open and free systems and programming languages. Such a system might be the basis for creating a “forked” solution (see Box 1) to fit the needs of those working on diseases of poverty in developing countries. In line with Oliveira and Salgado [12], we believe that a web-based solution to the complexities of running trials (especially multi-centre ones) and processing data is appropriate, as it reflects the information and technology expertise available globally that could be better used to support those engaged in clinical research. Other advantages of web-based systems are that they support simultaneous data entry from multiple sites and run using standard web browsers. Web-based technologies are rapidly being adopted in countries such as Kenya, where we are based. Here, as in many developing countries, there is a newly educated generation including, but not limited to, skilled computer scientists and informaticians, who will not only be passive recipients of such software but also become the future architects, developers, and maintainers of such systems. Individual researchers in any organisation should be able to more readily make use of such systems through standard information technology support provided by their employing organisation or institution.

**Box 1. An Introduction to Open-Source Software: Definitions and Required Reading**

1. Ten Things You Didn't Know about Open Source (http://www.tectonic.co.za/view.php?id=1465)
2. Definition of Open-Source Software
  - Free redistribution
  - Source code
  - Derived works
  - Integrity of the author's source code
  - No discrimination against persons or groups
  - No discrimination against fields of endeavour
  - Distribution of license
  - License must not be specific to a product
  - License must not restrict other software
  - License must be technology-neutral
  Taken from Opensource.org. See http://opensource.org/docs/definition.php for an annotated description of the above points.
3. “The Cathedral and Bazaar” by Eric S. Raymond (http://www.firstmonday.org/issues/issue3_3/raymond/). A seminal essay by a professional software developer that outlines his transition from open-source sceptic to advocate.
4. Fork: a substantial modification of a software system that takes it in a new direction. See http://en.wikipedia.org/wiki/Fork_(software_development) for more details.

## Conclusions

Although we perceive the need for the above-advocated approach to be most profound in developing countries and for those researchers working on small-to-large multi-centre non-commercial projects, if implemented correctly, its impact surely can only be beneficial to all clinical researchers. There are many examples of how open-source approaches have been used to assist scientific and biomedical research. Indeed, one eminent proponent of the open-source approach has even gone so far as to claim that PERL (an open-source language) saved the human genome project [13]. Another example that relates to medical research is Thomson International's well-known and widely used EndNote referencing software, which now relies on a commercial licensing of the power of the open-source MySQL database system. A good mainstream example might also support our point. Most of us, each and every day, utilise Web sites driven by the open-source Apache web server, which is the most common web server and has had over 50% of market share since 1998 [14].

Research organisations and funders should combine efforts to produce an open-source solution for trial data management. A shared platform could then be easily established, and would bring wider benefits such as electronic submission to regulators, automated sharing of data, and contribution to important public databases such as pharmacovigilance and drug-monitoring registries.

We believe that an open-source approach to a truly designed-for-purpose data-management system for clinical trials is attractive. Such a system would save money by eliminating the reliance on the use of expensive database software systems and their administrators. This would empower and enable a wider variety of people to conduct trials, as the question of capturing, cleaning, and extracting data would not be overly daunting or expensive. This point is significant, as it may encourage more investigators in resource-poor settings to take part in high-standard research that would otherwise be out of reach and beyond their capacity. Surely this would increase the scope and variety of trials that are conducted. Our hope for this article is that it will begin a debate on this topic, and lead to a concerted effort to lobby the international research and donor community to make sure this barrier to trial conduct is understood and addressed.
